# Speckle tracking-derived bi-atrial strain before and after eleven weeks of training in elite rowers

**DOI:** 10.1038/s41598-018-32542-8

**Published:** 2018-09-24

**Authors:** Mahdi Sareban, Kay Winkert, Billy Sperlich, Marc M. Berger, Josef Niebauer, Jürgen M. Steinacker, Gunnar Treff

**Affiliations:** 1 Institute of Sports Medicine, Prevention and Rehabilitation, Paracelsus Medical University, Salzburg, Austria; 2 Division of Sports and Rehabilitation Medicine, University Hospital Ulm, Ulm, Germany; 3 Institute of Sport Science, Integrative and Experimental Exercise Science and Training, University of Würzburg, Würzburg, Germany; 4 Department of Anesthesiology, Perioperative and General Critical Care Medicine, Salzburg General Hospital, Paracelsus Medical University, Salzburg, Austria

## Abstract

The left (LA) and right (RA) atria undergo adaptive remodeling in response to hemodynamic stress not only induced by endurance exercise but also as part of several cardiovascular diseases thereby confounding differential diagnosis. Echocardiographic assessment of the atria with novel speckle tracking (STE)-derived variables broadens the diagnostic spectrum compared to conventional analyses and has the potential to differentiate physiologic from pathologic changes. The purpose of this study was to assess and categorize baseline values of bi-atrial structure and function in elite rowers according to recommended cutoffs, and to assess the cardiac changes occurring with endurance training. Therefore, fifteen elite rowers underwent 2D-echocardiographic analysis of established variables of cardiac structure and function as well as STE-derived variables of bi-atrial function. Measurements were performed at baseline and after eleven weeks of extensive training. 40% of athletes displayed mildly enlarged LA and 47% mildly enlarged RA at baseline, whereas no athlete fell below the lower reference values of LA and RA reservoir strain. Average power during a 2000 m ergometer rowing test (P2000 m) improved from 426 ± 39 W to 442 ± 34 W (p = 0.010) but there were no changes of echocardiographic variables following training. In elite rowers, longitudinal bi-atrial strain assessment indicates normal resting function of structurally enlarged atria and thereby may assist to differentiate between exercise-induced versus disease-associated structural cardiac changes in which function is commonly impaired.

## Introduction

Endurance training is associated with hemodynamic alterations which, depending on type and intensity of exercise, lead to adaptive changes in cardiac structure and function. The cardiac adaptation in connection with endurance training are summarized in the term “athlete’s heart”^1^. A vast amount of data in athletes reveals a balanced structural enlargement of all cardiac chambers^1–3^ in connection with endurance exercise. Exercise-induced left (LA) and right (RA) atrial remodeling has recently received more scientific attention, because atrial fibrillation (AF) is more common in middle-aged athletes participating regularly in endurance events compared to non-athletes^4,5^. A complex interplay culminates in the increased prevalence of AF in individuals performing chronic intensified endurance exercise. Thereby, autonomic disturbances, atrial dilatation and possibly atrial fibrosis seem to play a decisive role^6^. In addition, current literature indicates that LA and RA are not only passive transport chambers but actively modulate ventricular filling and contribute to global cardiac performance^7^ via three repetitive functional phases, i.e. reservoir, conduit and contraction phase^7,8^. However, data on atrial function in athletes are scarce partly due to limitations of transthoracic echocardiography (TTE), the primary diagnostic tool for assessment of the atria^9^. In this regard echocardiographic B-Mode- and Doppler-derived analyses insufficiently extract intrinsic atrial function from ventricular interdependence and especially the assessment of distinct atrial phases. Recent advances in echocardiographic imaging, i.e. two-dimensional speckle tracking echocardiography (2D-STE)^10^, enables a more independent insight into atrial function by visualising and calculating each of the three phases separately^11^. Feasibility and reliability of speckle-tracking-derived atrial strain assessment depends on echocardiographic image quality improving automatic tracking of acoustic markers^12^ and cardiac sonographer/lab experience. In addition to the common superiority of echocardiographic image quality in athletes, our own data demonstrates good reliability of 2D-STE-derived bi-atrial strain assessment in different hemodynamic conditions^13^. LA reservoir strain cutoffs have even been suggested to catagorize the severity of ventricular diastolic dysfunction^14^ and risk evaluation of AF^15^. At the same time, RA reservoir strain has the potential to non-invasively evaluate the severity of right ventricular (RV) dysfunction^16^. Thus, 2D-STE-derived echocardiographic bi-atrial strain assessment seems promising as a non-invasive and reliable diagnostic tool for assessing atrial function and may assist to differentiate between exercise-induced versus disease-associated structural cardiac changes in which function is commonly impaired. Notably, existing echocardiographic studies on atrial structure and function in athletes i) are typically cross-sectional, ii) are restricted to the LA only, iii) do not assess all phases of atrial function independently and iv) have been examined only in a heterogeneous cohort of athletes in which physiologic variables as well as training volume and intensity differ^3,17^. This study aimed to investigate baseline values and the adaptive changes of established structural and functional echocardiographic variables as well as comprehensive bi-atrial STE-derived reservoir, -conduit and -contraction strain following an eleven weeks preparation period training in elite endurance athletes.

## Materials and Methods

### Study population

Fifteen rowers competing at national or international level participated in this prospective study which was originally designed to compare the effects of different training intensity distributions (for more details we refer to^18^). Table 1 summarizes the rowers’ anthropometric data.

**Table 1:** Participants’ (n = 15) physical characteristics.

Variable	Baseline	After eleven weeks	p	d_Cohen_
Age (years)	20 ± 3	21 ± 3	0.08	−0.08
Body height (cm)	188 ± 7.3	188 ± 7	0.91	−0.01
Body mass (kg)	88 ± 10	87 ± 10	0.01	0.10
Average power in 2000 m ergometer test (W)	426 ± 39	442 ± 34	0.01	−0.37
${\dot{{\rm{V}}}{\rm{O}}}_{2{\rm{\max }}}$ (mL/kg/min)	67 ± 6	67 ± 5	0.98	−0.03
Resting heart rate (bpm)	57 ± 7	56 ± 5	0.91	−0.02

Values are presented as arithmetic mean ± SD. bpm = beats per minute, ${\dot{{\rm{V}}}{\rm{O}}}_{2{\rm{\max }}}$ = Maximal oxygen consumption.

Prior to the study all participants provided written informed consent to participate. This study complied with the Declaration of Helsinki and its current amendments and the experimental protocol was approved by the ethical review board of the University of Ulm.

### Training intervention and documentation

Training was divided into four modes, namely (i) *Rowing*: boat and ergometer rowing, (ii) *Endurance*: other kinds of endurance training like running, cycling, swimming, etc., (iii) *Strength*: strength and resistance training, machine-based or weight lifting, and (iv) *Other*: stretching, stability training, etc. To quantify training volume and intensity, all rowers updated the mandatory online training diary of the German Rowing Federation daily. The diary included information about training mode, duration, distance, and intensity as well as information on days off and illness or injury. After completion of the study, all data were exported (.csv files) and subsequently analyzed.

### Pre- and post measurements

A licensed sports physician examined all rowers medically during the first visit and diagnosed all free from cardiovascular disease and eligible to take part in the study. The medical examination included electrocardiographic assessment at rest (CardioPart 12 Blue, Amedtec, Aue, Germany) and a comprehensive echocardiogram described in detail below. Afterwards, all conducted a series of rowing ergometer (Concept 2 Type D ergometer, Concept 2, Morrisville, USA) tests on two consecutive days. On the first day, maximal oxygen uptake (${\dot{{\rm{V}}}{\rm{O}}}_{2{\rm{\max }}}$; (Metamax 3x, Cortex Biophysics, Leipzig, Germany)) was measured during a ramp test protocol that enables a linear increase in power and objective test termination^19^. ${\dot{{\rm{V}}}{\rm{O}}}_{2}$ was averaged over 30-second intervals and ${\dot{{\rm{V}}}{\rm{O}}}_{2{\rm{\max }}}$ was defined as the highest ${\dot{{\rm{V}}}{\rm{O}}}_{2}$ despite increasing workload. On the second day, all rowers performed an all-out 2000 m ergometer test to evaluate maximal rowing ergometer performance, by covering the virtual distance of 2000 m on a rowing ergometer as fast as possible. The average and maximal power determined changes in performance^20,21^.

Hydration status was assessed from hematocrit (Htc) and hemoglobin concentration [Hb] from venous blood samples taken in the morning of the examinations to account for the impact of intravascular volume status of echocardiographic variables. Changes in blood and plasma volume (%ΔPV) were estimated employing the equation by Dill and Costill^22^.

### Echocardiographic assessment

All rowers underwent transthoracic echocardiography using a commercially available ultrasound system (Philips CX50, Phillips Medical Systems, Andover, MA, USA) with a 1.0–5.0 MHz sector array transducer (Philips S5-1, Phillips Medical Systems, Andover, MA, USA) at baseline and after the eleven weeks of preparation. The same experienced cardiac sonographer performed all acquisitions the day prior to ${\dot{{\rm{V}}}{\rm{O}}}_{2{\rm{\max }}}$-testing with the individual lying in left lateral decubitus position according to current guidelines^23^. An electrocardiogram connected to the ultrasound system recorded heart rate during the examination. All images were saved on a mass storage device in raw Digital Imaging and Communications in Medicine format and analyzed offline using commercially available software (Philips Xcelera, Phillips Medical Systems, Andover, MA, USA).

According to the latest recommendations about normal values of cardiac chambers in adults (as proposed by the the American Society of Echocardiography and the European Association of Cardiovascular Imaging (ASE/EACVI)^23^), severity partition cutoff values only exists for left ventricular (LV) ejection fraction (EF), -size and -mass as well as LA maximum volume. For other parameters, the mean value plus 1.96-fold standard deviation (SD) of gender-, age-, and body surface area (BSA)–normalized parameters classify the upper limit of normal and were applied as cutoff values to analyze the echocardiographic results of the present study. We used the upper cutoff value of normal LV EDV indexed to BSA (i.e. >74 ml/m²) to identify participants with classic feature of exercise-induced cardiac remodeling in order to perform group comparison of our strain results. Reference values for LA and RA reservoir strain were employed according to current recommendations^24,25^.

### 2D echocardiography

Image quality optimization, linear, and volumetric measurements of cardiac chambers were performed according to the current recommendations. Briefly, LV and LA volumes were assessed using biplane disk summation algorithm by manually tracing endocardial borders in the apical 4- and 2-chamber views. When tracing the LA borders, the confluences of the pulmonary veins were excluded, and the atrioventricular interface was represented by the mitral annulus plane. LA maximal volume was measured just before the opening of the mitral valve. RV areas were measured by manual tracing of RV endocardial border. RA volumes were measured using the single-plane (apical 4-chamber) method of disks.

The echocardiographic report contains all linear and volumetric measurements in absolute numbers as well as indexed to BSA calculated using the formula by Mosteller^26^.

### Conventional doppler, tissue doppler imaging and M-Mode

Conventional Doppler and Tissue-Doppler Images (TDI) were obtained from the apical 4-chamber view. For assessment of transmitral flow, the pulsed-wave Doppler sample volume was placed between the mitral leaflet tips in order to obtain peak early (E) and late diastolic (A) flow velocities. For TDI assessment of peak early diastolic (e’), late diastolic (a’) and systolic (s’) LV excursion velocities ultrasound beam was aligned to the longitudinal motion of the LV and the pulsed-wave sample volume placed at the basal septum and basal lateral wall. Peak velocities from the septal and lateral side of mitral annulus were averaged. e’, a’, s’ of RV excursion velocity were assessed by placing the pulsed-wave TDI sample volume in the tricuspid annulus of the RV free wall from a standard apical 4-chamber window^27^. Tricuspid annular plane systolic excursion (TAPSE) was assessed by placing an M-mode beam through the lateral tricuspid annulus^27^. The myocardial performance index (MPI) was calculated as the ratio of isovolumetric time divided by ejection time and derived from TDI analyses^27^.

### Two-dimensional myocardial speckle tracking echocardiography

**Figure 1:**
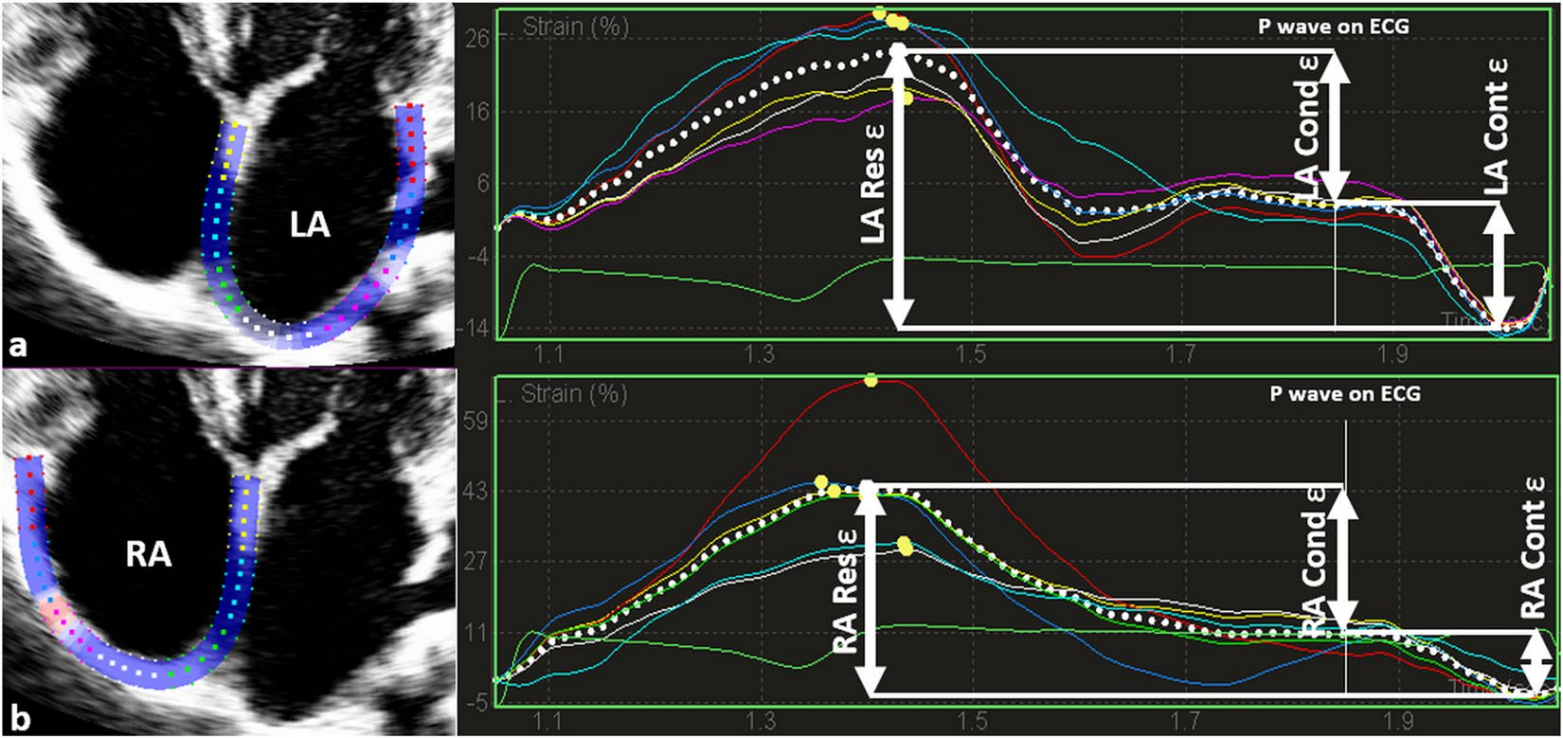
LA (a) and RA (b) strain (ε) curve using QRS-timed analysis. Dotted lines depict average curve of the six segments. The first positive peak of the curve is the peak atrial ε during ventricular systole, measured at the end of the reservoir phase (LA Res ε). The peak deflection is followed by a plateau and peak atrial ε in late diastole at the onset of the P wave on the electrocardiogram, just before the active atrial contraction (LA Cont ε) begins. La conduit (LA Cond ε) is calculated as the difference between LA Res ε and LA Cont ε.

Longitudinal STE-derived LV and LA strain measurements were obtained from the apical 4- and 2-chamber views whereas RA strain was obtained from apical 4-chamber views. The researcher optimized image quality by positioning the focus at the region of interest and adjusting sector depth and width to include as little as possible outside the region of interest in order to maintain a frame rate between 50–80/sec. The same researcher performed offline analyses using a commercially available acoustic tracking software package (QLAB 9 (cardiac motion quantification (CMQ)); Phillips Medical Systems, Andover, MA, USA). Thereby, the region of interest was set at the myocardium using a point-and-click technique and the software divided the atrial wall into six equidistant segments. Orifices of the pulmonary venous component and segments in which inadequate tracing was observed were excluded from further analyses and the remaining segments were averaged. According to current practice recommendations^28^ and depending on the software package used in this study, LA reservoir, -conduit and -contraction strains were calculated with the first reference frame set at the onset of the QRS-wave of the surface ECG as illustrated in Fig. 1. Values obtained from apical 4- and 2-chamber views were averaged to obtain bi-plane results.

### Statistical analysis

All statistical analyses were performed using SPSS 21 for Windows (SPSS, Inc., Chicago, IL) with continuous variables presented as arithmetic mean ± SD and categorical variables as percentages. Student paired *t* test compared pre- and post-protocol measurements after confirmation of normal distribution using the Shapiro-Wilk-test. A p-value ≤ 0.05 was considered to be statistically significant. Cohens d (d_Cohen_) was calculated to estimate effect sizes with the thresholds for small, moderate, and large effects set at 0.20, 0.50, and 0.80, respectively^29^. Pearson’s r coefficient of correlation assessed correlations between variables.

The datasets analyzed during the study are available from the corresponding author on reasonable request.

## Results

### Training and performance

The average training volume was 961 ± 82 min/week with 539 ± 77 min per week rowing, 163 ± 55 min per week strength training, 173 ± 59 min per week with endurance training and 84 ± 32 min other training activities. The power during the 2000 m all-out ergometer test improved significantly (+3.8%; p = 0.010) after eleven weeks of preparation, whereas ${\dot{{\rm{V}}}{\rm{O}}}_{2{\rm{\max }}}$ did not change from pre to post testing (Table 1).

### Standard structural and functional LV parameters

Structural and functional LV parameters at baseline and after eleven weeks of training are summarized in Table 2. According to the current ASA/EACVI cut-offs, defined as 103–116 g/m² the rowers’ mean BSA-indexed LV mass at baseline was categorized as mildly abnormal. BSA-indexed LV end-diastolic volume at baseline was close to the threshold between normal and mildly abnormal (75–89 mL/m²) according to the current ASA/EACVI cutoffs. 60% of the athletes presented mildly enlarged LV during at least one echocardiographic examination. Sub-group analysis between participants with enlarged LV and participants who do not feature this classic sign of exercise-induced LV remodeling, did not elicit a significant difference in LV strain results (p = 0.284). There was no significant change of morphologic or functional LV parameters between baseline and after eleven weeks of training (Table 2). There was no change in %ΔPV (1.2 ± 5.6%; p = 0.47).

**Table 2:** Morphologic and functional LV indices at baseline and after eleven weeks of training in n = 15 elite rowers.

Variable	Baseline	After eleven weeks	p	d_Cohen_
**Morphologic indices**				
LV EDD (cm)	5.4 ± 0.4	5.4 ± 0.3	0.99	0.01
LV EDD/BSA (cm/m²)	2.5 ± 0.2	2.5 ± 0.2	0.61	0.09
LV EDV (mL)	158.3 ± 26.6	144.9 ± 23.3	0.07	0.52
LV EDV/BSA (mL/m²)	74.2 ± 12.8	68.8 ± 13.0	0.09	0.49
LV ESV (mL)	67.7 ± 14.8	63 ± 15.4	0.29	0.31
LV ESV/BSA (mL/m²)	31.7 ± 6.6	29.7 ± 7.2	0.37	0.34
LV Mass (g)	238.2 ± 35.2	247.3 ± 35.0	0.11	−0.32
LV Mass/BSA (g/m²)	111.1 ± 13.3	116.2 ± 14.0	0.07	−0.37
**Functional indices**				
LV ejection fraction (%)	57.7 ± 4.7	55.6 ± 3.6	0.13	−0.49
LV longitudinal strain (%)	18.8 ± 2.0	18.4 ± 3.0	0.49	−0.14
LV stroke volume (mL)	90.6 ± 16.2	81.9 ± 14.9	0.07	−0.85
E (cm/s)	80.6 ± 14.8	73.4 ± 17.7	0.10	0.44
A (cm/s)	47.5 ± 12.3	42.2 ± 8	0.12	0.50
E/A ratio	1.8 ± 0.3	1.8 ± 0.4	0.87	−0.04
av e’ (cm/s)	15.9 ± 2.0	15.1 ± 2.1	0.10	0.48
av a’ (cm/s)	6.9 ± 1.1	7.3 ± 1.5	0.37	−0.33
E/av e’ ratio	5.1 ± 0.6	4.9 ± 0.9	0.42	0.18
av s’ (cm/s)	10.6 ± 1.9	10.4 ± 1.9	0.73	0.08

Values are presented as arithmetic mean ± SD. EDD = end-diastolic diameter, EDV = end-diastolic volume, ESV = end-systolic volume, BSA = body surface area, E = pulsed-wave 2D-imaging-derived peak early transmitral diastolic filling velocity, A = pulsed-wave 2D-imaging-derived peak late transmitral diastolic filling velocity, e’ = pulsed-wave Doppler tissue imaging (DTI)-derived peak early diastolic myocardial velocity (averaged from basal septum and basal lateral LV wall), a’ = pulsed-wave Doppler tissue imaging (TDI)-derived peak late diastolic myocardial (tissue) velocity (averaged from basal septum and basal lateral LV wall), s’ = pulsed-wave Doppler tissue imaging (DTI)-derived peak systolic myocardial velocity (averaged from basal septum and basal lateral LV wall).

### Standard structural and functional RV parameters

Structural and functional RV parameters are summarized in Table 3.

**Table 3:** Morphologic and functional RV indices at baseline and after eleven weeks of training in n = 15 elite rowers.

Variable	Baseline	After eleven weeks	p	d_Cohen_
**Morphologic indices**				
RVOT proximal (cm)	3.3 ± 0.4	3.3 ± 0.6	0.52	0.19
RVOT proximal/BSA (cm/m²)	1.5 ± 0.2	1.6 ± 0.3	0.57	0.12
RV EDA (cm²)	30.5 ± 6.2	30.7 ± 4.8	0.89	−0.03
RV EDA/BSA	14.3 ± 2.9	14.4 ± 1.9	0.87	−0.04
RV ESA (cm²)	16.5 ± 5.3	17.0 ± 3.5	0.67	−0.12
RV ESA/BSA (cm²/m²)	7.7 ± 2.5	8.0 ± 1.5	0.61	0.10
**Functional indices**				
TAPSE (mm)	24.9 ± 2.4	23.7 ± 2.8	0.11	0.47
RV FAC (%)	46.5 ± 9.1	44.8 ± 5.5	0.55	0.23
Tissue Doppler MPI (%)	0.4 ± 0.1	0.4 ± 0.1	0.66	−0.12
s’ (cm/s)	13.8 ± 2.0	14.0 ± 2.5	0.82	−0.06
e’ (cm/s)	13.1 ± 3.0	13.8 ± 2.8	0.52	−0.22
a’ (cm/s)	8.4 ± 2.5	8.7 ± 2.7	0.65	−0.12

Values are presented as arithmetic mean ± SD. RVOT = right-ventricular outflow tract from parasternal short axis view; EDA = end-diastolic area, ESA = end-systolic area; BSA = body surface area, TAPSE = Tricuspid annular plane systolic excursion, FAC = Fractional area change, MPI = myocardial performance index, s’ = pulsed-wave Doppler tissue imaging (DTI)-derived peak systolic myocardial velocity (averaged from basal septum and basal lateral RV wall), e’ = pulsed-wave Doppler tissue imaging (DTI)-derived peak early diastolic myocardial velocity (averaged from basal septum and basal lateral RV wall), a’ = pulsed-wave Doppler tissue imaging (TDI)-derived peak late diastolic myocardial (tissue) velocity (averaged from basal septum and basal lateral RV wall).

Using the current ASA/EACVI recommendations of normal values for cardiac chamber quantification in adults, absolute as well as indexed mean RV areas exceeded upper reference limits whereas mean linear RV dimensions remained within normal range. 86.7% of athletes presented with BSA-indexed end-diastolic areas above the upper reference limit (defined as greater than or equal to 12.6 cm²/m²) during baseline echocardiographic examination. Reduction in RV function was not detected using the reference values from the ASE/EACVI (i.e., FAC < 33%; TAPSE < 17 mm; s’ < 6 cm/sec). No significant change of structural or functional parameters occurred between baseline and after eleven weeks of training (Table 3).

### Structural and functional LA parameters

Structural LA parameters are listed in Table 4.

**Table 4:** LA and RA morphologic indices at baseline and after eleven weeks of training in n = 15 elite rowers.

Variable	Baseline	After eleven weeks	p	d_Cohen_
**Left atrium**				
AP dimension (cm)	3.8 ± 0.4	3.7 ± 0.5	0.26	0.27
AP dimension/BSA (cm/ m²)	1.8 ± 0.2	1.7 ± 0.3	0.36	0.19
Biplane max volume (mL)	69.5 ± 17.2	63.3 ± 17.3	0.08	0.36
Biplane max volume/BSA (mL/m²)	32.6 ± 7.8	30.0 ± 7.8	0.09	0.34
**Right atrium**				
Minor axis dimension (cm)	4.5 ± 0.6	4.7 ± 0.7	0.96	−0.06
Minor axis dimension/BSA (cm/m²)	2.2 ± 0.3	2.2 ± 0.3	0.77	−0.07
Major axis dimension (cm)	5.4 ± 0.4	5.5 ± 0.3	0.59	−0.14
Major axis dimension/BSA (cm/m²)	2.5 ± 0.3	2.6 ± 0.3	0.57	−0.12
Max volume (mL)	74.0 ± 16.8	74.3 ± 18.9	0.94	− 0.02
Max volume/BSA (mL/m²)	34.5 ± 7.3	34.9 ± 8.7	0.86	− 0.05

Values are presented as arithmetic mean ± SD. AP = anteroposterior, BSA = body surface area.

**Figure 2:**
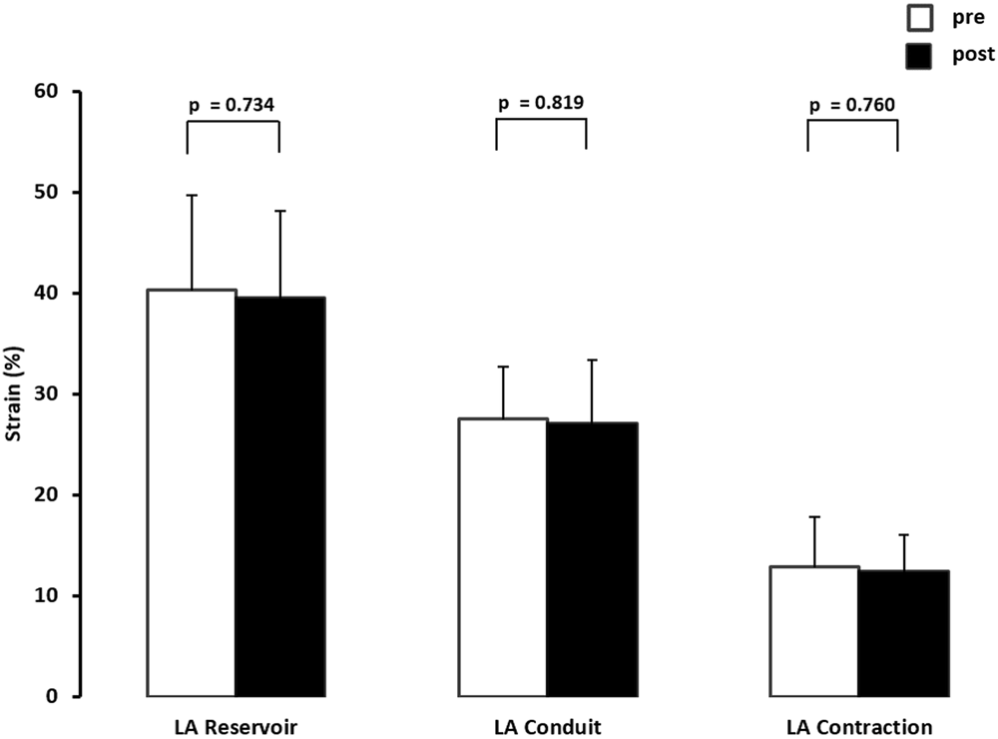
Changes of left atrial (LA) reservoir, -conduit, and -contraction speckle tracking-derived strain of n = 15 elite rowers before (pre) and following eleven weeks of training (post).

Using the current ASA/EACVI recommendations of normal values for cardiac chamber quantification in adults, mean LA dimension and volumes were within normal range. 40% of athletes revealed mildly enlarged LA (defined as indexed volume greater than or equal to 35 mL/m²) at baseline whereas no athlete fell below the lower reference value for LA reservoir strain. There was no significant change of structural atrial parameters between baseline and after eleven weeks of training (Table 4). Sub-group analysis between participants with enlarged (>74 ml/m² BSA) end-diastolic LV and participants who do not feature this classic sign of exercise-induced LV remodeling, did not elicit a significant difference in LA and RA strain results. Image quality for STE-derived strain assessment was good in all subjects, however, 6 of 180 possible LA-segments had to be excluded due to inadequate speckle tracking. LA reservoir, -conduit and -contraction strain did not change from pre to post testing (Fig. 2).

### Morphologic and functional RA parameters

**Figure 3:**
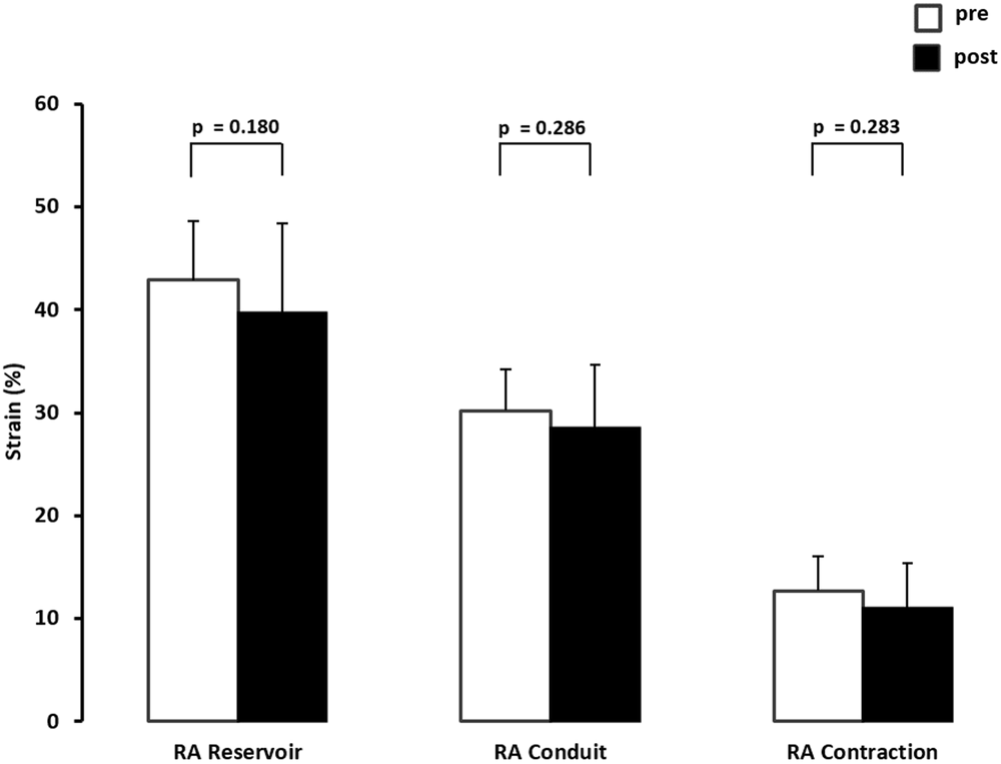
Changes of right atrial (RA) reservoir, -conduit, and -contraction speckle tracking-derived strain of n = 15 elite rowers before (pre) and following eleven weeks of training (post).

Structural RA parameters are listed in Table 4. Using the current ASA/EACVI recommendations of normal values for cardiac chamber quantification in adults, mean RA dimension and volumes were within normal range. 47% of the athletes revealed mildly enlarged RA (defined as indexed volume greater than or equal to 39 mL/m²) at baseline whereas no athlete fell below the lower reference value for RA reservoir strain. There was no significant change of structural or standard functional atrial parameters between baseline and after eleven weeks of training (Table 4). Image quality for STE-derived strain assessment was good in all subjects and all 180 RA-segments were employed for further analysis. RA reservoir, -conduit and -contraction strain did not change between pre and post testing (Fig. 3).

### Correlation analysis

LA reservoir strain did not correlate significantly with maximal LA volume (r = −0.10; p = 0.61) or LV end-diastolic volume (r = −0.03; p = 0.88). Likewise, RA reservoir strain did not correlate with maximal RA volume (r = 0.13; p = 0.50) and RV end-diastolic area (r = −0.04; p = 0.85).

Change in average 2000 m ergometer test power output did not correlate significantly with change in LA reservoir (r = −0.38; p = 0.25), -conduit (r = −0.37; p = 0.27) and -contraction strain (r = −0.26; p = 0.43) nor with change in RA reservoir (r = −0.02; p = 0.96), -conduit (r = 0.38; p = 0.25) and -contraction strain (r = −0.13; p = 0.70). Change in ${\dot{{\rm{V}}}{\rm{O}}}_{2{\rm{\max }}}$ did not correlate significantly with change in LA reservoir (r = 0.14; p = 0.64), -conduit (r = 0.15; p = 0.96) and -contraction strain (r = 0.26; p = 0.39) nor with change in RA reservoir (r = 0.20; p = 0.52), -conduit (r = 0.26; p = 0.39) and -contraction strain (r = 0.07; p = 0.82).

## Discussion

The present study shows that (i) approximately half of our elite rowers exhibited bi-atrial enlargement while STE-derived bi-atrial strain parameters were within normal limits, (ii) no structural or functional adaptation occurred after eleven weeks of preparation training, (iii) the vast majority of athletes exceeded internationally accepted upper reference limits of RV enlargement.

Rowing is unique within the broad spectrum of endurance disciplines, as it opposes the athlete not only to high isotonic but also exceptional high isometric cardiac stress, which are both important mediators for exercise-induced cardiac remodelling. Furthermore, endurance disciplines characterized by high concomitant isotonic and isometric stress appear to result in different and more pronounced structural and functional LV remodeling patterns than disciplines with more isolated isotonic stimuli, as distance running or soccer^30^. The average training volume of our athletes was 961 ± 82 min/week with 539 ± 77 min per week rowing and 163 ± 55 min per week strength training, pointing out the enormous exposure of our elite rowers to cardiac stress. Thus, elite rowers offer a unique opportunity to study new and evolving variables of cardiac structure and function assessment which might assist improving the differentiation between exercise-induced adaptive remodeling and cardiac pathology. Noteworthy, 40% of the athletes in our study exceeded the upper ASE/EACVI reference limit of normal LA volume (i.e. 34 mL/m²). In case of concurrent clinical symptoms as dyspnea, which is a common indication for which athletes are referred to echocardiography, their presumably adaptive atrial enlargement overlaps with atrial enlargement seen in different pathologic heart conditions.

Assessment of functional atrial parameters has become feasible since the advent of STE and holds promise to guide the diagnostics within this uncertain atrial gray zone. D’Ascenci^31^ showed significantly reduced STE-derived LA reservoir (39.7 ± 8.4%) and -contraction (10.6 ± 3.8%) strains in healthy competitive adolescent male soccer players^31^ but not in female volleyball players (LA reservoir: 43.9 ± 9.5%; contraction strain: 15.5 ± 4.0%)^17^. Our study extends these findings towards a cohort of healthy elite male rowers. Although we did not directly compare our athletes with a control group, our results (LA reservoir strain: 43.1 ± 5.3%) are in line with the mean LA reservoir strain from a large cohort of healthy non-athletic individuals (45.1 ± 11.4%)^24^ despite the pronounced adaptive hemodynamic stimulus during rowing. Noteworthy, the recent recommended lower reference value for LA reservoir strain is 22.7%^24^ and none of our athletes fell below this value. Though slightly reduced LA reservoir strain at rest may be caused by exercise-induced adaptive changes of heart function^17^, its assessment has the potential to support the diagnostic work-up in athletes with structural LA changes. Reference values for LA conduit and -contraction strain are not published yet and thus no comparison can be provided.

In addition, this study extends two longitudinal-designed studies assessing STE-derived LA strain in team-sports athletes^17,32^. D’Ascenzi showed a significant reduction of LA reservoir and -contraction strain after eight months training in male soccer players as well as after 16 weeks of training in female volleyball players. In contrast, none of the phasic LA strain parameters changed during eleven weeks of intensive preparation period training in our male rowers. These findings have important clinical implications, considering that middle-aged males who have been engaged in strenuous endurance training for more than 10 years and who are otherwise healthy, are at risk of developing AF^5^. Taking into account the overall health benefits and even reduced cardiovascular disease prevalence in former elite rowers^33^ continuing athletic lifestyle while reducing the risk of AF has the potential to further improve cardiovascular health of endurance athletes. In light of the reduced STE-derived LA strain in individuals with paroxysmal AF^34^, and considering the preserved resting strain results despite large volume of training in our study, monitoring LA function in endurance athletes has the potential to detect a drift in strain to evaluate the risk and induce early preventive strategies of AF in endurance athletes.

In parallel to the LA, the RA faces similar diagnostic uncertainties when assessing only structural parameters in athletes with clinical symptoms. In our study, 47% of athletes showed enlarged RA using the current reference values from the ASE/EACVI. RA reservoir strain at rest is reduced by 27.1 ± 11.6% in patients with pulmonary hypertension and the reduction is thought to reflect RV dysfunction^16,35^, which is a common complication of arrhythmogenic right ventricular cardiomyopathy (ARVC)^36^. Thus STE-derived RA strain might serve as a diagnostic aid to differentiate between a potentially life-threatening condition and physiologic, exercise-induced RV adaptation. The recently recommended lowest expected value for RA reservoir strain is 24%^25^ and no athlete in our study was below this value. In addition, our STE-derived RA strain results (Reservoir strain: 42.9 ± 5.7%) are in agreement with data by Pagourelias *et al*.^3^ who did not observe any difference of RA reservoir and –contraction strain between three groups consisting of endurance athletes (Reservoir strain: 43.9 ± 15%), athletes from game sports, and a control group. Phasic RA strain parameters did not change during eleven weeks of intensive preparation period training in our study what is in line with the only longitudinal study investigating the impact of training on RA strain in female volleyball players^17^. Our study extends previous results^17^ with male endurance athletes and some trials concluded an even more modest atrial adaptation to training in women^37^. In parallel to our LA results, our data suggest that STE-derived resting RA strain assessment is no appropriate surrogate for exercise-induced atrial adaptation but has the potential to support diagnostic work-up in athletes with symptoms and suspected right heart disease.

In addition to the abovementioned structural atrial changes, 60% of our athletes yielded enlarged LV and 94% enlarged RV according to the current reference values. RV dilatation is a common phenotypic expression in ARVC, a disease in which competitive sports participation is a modifiable risk factor for sudden cardiac death^38^. Notably, 67% of athletes in our study fulfilled the minor ARVC criterion for BSA-indexed RV dilatation whereas none fulfilled the major ARVC criterion^39^. Recently new cutoffs derived from a large cohort of top-level athletes from different sports were published^40^ and 20% of our athletes even exceeded the suggested upper reference value (i.e. 36 mm RV Outflow Diameter in the short axis view for male athletes). In line with the atrial results, reduction in RV function was not found in our cohort of elite endurance athletes. 53.5% even exhibited supranormal systolic RV function at baseline as indicated by an s’ > 13.4 cm/sec. Concerning the diagnostic gray zone between exercise-induced RV remodeling and ARVC as well as concerns about potential myocardial RV damage due to intense endurance exercise, the major ARVC Task Force criteria or reference values derived from an athletic population in combination with functional assessment should be employed in the interpretation of echocardiographic-derived structural RV parameters in endurance-trained athletes.

Our study has limitations worth indicating. From a methodological standpoint a larger sample size may be favorable. However, we aimed to investigate a selected cohort of elite endurance athletes competing at national and international level. Therefore, the number of subjects is limited by the nature of the highly selective study cohort. Adding more individuals into the study would have meant to integrate weaker athletes which were not the target of the study and with more athletes the individual monitoring of training load becomes more challenging. No follow up examinations were performed because athletes were competing in international races making group testing of all athletes logistically impossible. This study was conducted with male athletes only. Sex significantly affects the normal heart size in athletes^41^. Thus, the results of this study cannot be transferred to female endurance athletes.

In summary, this study has shown that mildly enlarged LA and RA maximal volumes are common in elite rowers whereas STE-derived resting bi-atrial strain variables are within normal limits and do not change after eleven weeks of training. Hence, variables of atrial strain at rest are unsuitable to evaluate exercise-induced atrial remodelling. but have the potential to guide the diagnostic work-up in symptomatic athletes with structural atrial changes. Furthermore, considering the stable echocardiographic variables despite large volume of endurance training, our study further supports the cardiac safety of competitive rowing.
